# 3D Bioprinting of Carboxymethylated-Periodate Oxidized Nanocellulose Constructs for Wound Dressing Applications

**DOI:** 10.1155/2015/925757

**Published:** 2015-05-19

**Authors:** Adam Rees, Lydia C. Powell, Gary Chinga-Carrasco, David T. Gethin, Kristin Syverud, Katja E. Hill, David W. Thomas

**Affiliations:** ^1^Welsh Centre for Printing and Coating (WCPC), Swansea University, Swansea SA2 8PP, UK; ^2^Centre for NanoHealth, Swansea University, Swansea, UK; ^3^Tissue Engineering and Reparative Dentistry, Cardiff University School of Dentistry, Cardiff CF14 4XY, UK; ^4^Paper and Fibre Research Institute (PFI), Høgskoleringen 6b, 7491 Trondheim, Norway

## Abstract

Nanocellulose has a variety of advantages, which make the material most suitable for use in biomedical devices such as wound dressings. The material is strong, allows for production of transparent films, provides a moist wound healing environment, and can form elastic gels with bioresponsive characteristics. In this study, we explore the application of nanocellulose as a bioink for modifying film surfaces by a bioprinting process. Two different nanocelluloses were used, prepared with TEMPO mediated oxidation and a combination of carboxymethylation and periodate oxidation. The combination of carboxymethylation and periodate oxidation produced a homogeneous material with short nanofibrils, having widths <20 nm and lengths <200 nm. The small dimensions of the nanofibrils reduced the viscosity of the nanocellulose, thus yielding a material with good rheological properties for use as a bioink. The nanocellulose bioink was thus used for printing 3D porous structures, which is exemplified in this study. We also demonstrated that both nanocelluloses did not support bacterial growth, which is an interesting property of these novel materials.

## 1. Introduction

Nanocellulose is a novel material that can be produced from a variety of biodegradable and renewable resources and holds the potential to transform various areas of research and development [1–7]. As a novel biomaterial, nanocellulose may be deposited as a gel to form three-dimensional structures that have potentially diverse applications. The material has various characteristics that make it suitable as a substrate for printing functionality and also as a component in bioinks. Firstly, well-fibrillated nanocellulose materials are composed of a large fraction of nanofibrils with widths in the nanometer scale and lengths in the micrometer scale. These high aspect ratio nanofibrils are capable of self-assembling and form dense, smooth, transparent, and strong structures [8, 9]. Based on these characteristics, recent studies have proposed using nanocellulose as the main component in the production of smooth films, which are suitable for subsequent functionalization by printing [10, 11].

Secondly, depending on the pretreatment, different types of nanocellulose can be produced with a defined morphology and surface chemistry. In this respect, it has been demonstrated that oxidized nanocellulose gels could act as pH responsive structures, based on cellulose nanocrystals [12] or cellulose nanofibrils [13], which may have applications in dressings for chronic wounds. The development of novel biomaterial solutions with added functionality is of great interest in wound healing research, as current medical treatments present major societal, economic, and clinical challenges. Recently, the cytotoxicity of various nanocellulose qualities has been verified, confirming that the assessed materials were not cytotoxic against a series of cell lines [14–16]. This gives additional and supportive evidence with respect to the suitability of the material for use as a medical device in wound healing.

The design of tailor-made wound dressings with barrier, absorbent, and bioresponsive characteristics may benefit from the assembly of complex porous structures in an effective way. Printing and coating are low-cost processes for surface modification and can facilitate the deposition of specific nanocellulose materials. Provided that nanocellulose can be applied as a bioink, printing can be an effective method to form self-standing and high-absorbent porous structures.

The deposition of gelling materials can be challenging. Traditional volume printing methods are candidate processes, when the material is in a liquid state. At the onset of gelation, the image transfer process breaks down and alternative technologies that use an extrusion principle are more appropriate. These rely on the ability to maintain constant rheological properties within the gel combined with a preparation process that ensures gel consistency to facilitate volume production.

Deposition of gel materials can be achieved using a Bioplotter [17–21]. Bioplotting is considered to be very similar to a variant of rapid prototyping with the exception of the method of deposition. Some variants of rapid prototyping use a mechanical screw and heat source to melt a solid polymer and then extrude it as a highly viscous fluid through a nozzle, whereas the Bioplotter uses air pressure to extrude, for example, gels. This method is appropriate for low viscosity fluids, including polymer melts and biopolymers, such as nanocellulose gels. This delivery system has several advantages in that liquids having an appropriate viscosity can be deposited using the Bioplotter. The Bioplotter can also produce complex shapes that would otherwise be unfeasible through traditional manufacturing techniques.

This paper describes how nanocellulose gels may be deposited using a Bioplotter that is capable of extruding the gel to form a predefined 3D structure. The constructs are “fixed” by freeze-drying, after which their topographical details are explored using microscopy techniques. The study demonstrates that gel structures can be printed on nanocellulose films to “pattern” the surface. Additionally, nanocellulose films have characteristics which could allow for potential use as a wound dressing material. Therefore, we studied the ability of these prototypical nanocellulose structures to inhibit bacterial growth. 

## 2. Materials and Methods

### 2.1. Nanocellulose Production

Two nanocellulose materials have been applied in this study, both derived from* Pinus radiata* bleached kraft pulp fibers. One nanocellulose material was pretreated with TEMPO mediated oxidation [1, 5] and will be denominated as TEMPO nanocellulose in the following text. The second nanocellulose material was pretreated with a combination of carboxymethylation and periodate oxidation [13] and will be described as C-Periodate nanocellulose in the following text. The nanocelluloses were produced through homogenization using a Rannie 15 type 12.56X homogenizer. The pulp consistency was estimated by measuring the dry residue with an HR73 Moisture Analyzer. The consistency of the TEMPO and C-Periodate samples was 1.0% and 0.5%, respectively.

As an attempt to remove residual fibers from the C-Periodate nanocellulose material, the suspension was additionally filtered through a Sefar 03-140/41 mesh fabric (mesh opening = 140 *μ*m). The filtered nanocellulose was then concentrated by centrifugation for 30 min at 4000 rpm. The final consistency of the C-Periodate nanocellulose was 3.9%. This sample was used as bioink for bioprinting.

### 2.2. Characterisation

The content of carboxyl and aldehyde groups for the nanocellulose materials was quantified by conductometric titration, as described by [13, 22]. The degree of polymerization (DP) was calculated from intrinsic viscosity values according to the standard ISO 5351:2010 [23].

Films (20 g/m^2^) made of the TEMPO and C-Periodate nanocelluloses were used to estimate the amount of residual fibers that were not fibrillated into nanofibrils. The films were made by casting a 0.2% suspension in Petri dishes. The suspensions were allowed to dry for 4-5 days at room temperature (~20°C). Following drying, the substrates were characterized fully by using three techniques: laser profilometry (LP), scanning electron microscopy (SEM), and atomic force microscopy (AFM). These were chosen because they complement the structural characterization by covering the quantification of features from the micro- to the nanoscale.

Ten topographical images were acquired from each sample by LP. The topside was assessed. The size of the images was 1 mm × 1 mm, with a resolution of 1 *μ*m. The roughness of the films was quantified and the amount of residual fibers was estimated based on an updated model reported by Chinga-Carrasco et al. [24].

SEM imaging was performed with a Hitachi S-3000 variable pressure SEM, using a secondary electron detector. The acceleration voltage was 5 kV and the working distance was approximately 10 mm.

AFM was used for assessing nanofibril morphology. AFM imaging of the two nanocellulose materials was performed using a Dimension 3100 AFM (Bruker) and RTESPA probes (Bruker). Imaging was performed in tapping-mode operation in air, using a scan rate of 0.7 Hz and an image size of 1024 × 1024 pixels.

The rheological properties of the materials were measured using a Bohlin Gemini HR nano Rheometer (Malvern Instruments Ltd., UK), in simple shear mode. The tests were conducted using a plate and cone arrangement. A 4°, 40 mm diameter cone was used and the rheometer had a set gap of 0 mm. The test was run under ambient conditions, increasing the strain rate from 1 s^−1^ to 1000 s^−1^ and then decreasing the rate.

### 2.3. Bacterial Growth Assessment

The nanocellulose materials were diluted in deionized water to 0.4% and screened for bacterial contamination. This was performed by incubating the nanocellulose suspensions at 37°C in an aerobic environment and taking hourly optical density measurements at 600 nm (OD_600_) of the samples over 17 hrs. A Mueller Hinton (MH) broth only control was also tested. In addition, the ability of the nanocellulose materials to inhibit or promote bacterial growth in planktonic culture was also examined. Nanocellulose suspensions (800 *μ*L) were inoculated with 20 *μ*L of* Pseudomonas aeruginosa* PAO1 overnight culture and hourly OD_600_ measurements taken as described above. An MH broth control, comprising nanocellulose material (800 *μ*L) with 20 *μ*L MH broth, was also included.

### 2.4. Three-Dimensional (3D) Bioprinting

A commercially available 3D Bioplotter (EnvisionTEC GmbH) was used to construct two different structures from the nanocelluloses. The shear rate in the printer was of the order 2000/s through the nozzle. The first construct was a grid structure deposited on the cast film and the second was a 3D structure comprising nine layers to explore the possibility to form porous scaffolds. Each layer in the 3D printed structure consisted of 12 × 12 tracks. The TEMPO nanocellulose was consistently used as substrate and the C-Periodate nanocellulose was used as the bioink. Calcium chloride (CaCl_2_, 0.05 M) was pipetted on to some of the layered constructs to fix the material by ionic cross-linking. The 3D printed constructs were then freeze-dried and assessed visually and also by SEM imaging.

## 3. Results and Discussion

The two nanocelluloses differed greatly with respect to morphology and surface chemistry. Note the major difference with respect to DP between the TEMPO and C-Periodate nanocellulose materials (Table 1). This was an indication of distinct nanofibril length [25]. Additionally, TEMPO mediated oxidation introduced a significant amount of carboxyl groups and a small amount of aldehydes in the material. The carboxymethylated and periodate oxidized sample possessed a significant amount of both aldehyde and carboxyl groups [13]. The surface chemistry of these materials was most interesting as the functional groups could be used for biosensing capabilities and for cross-linking, which also could improve the mechanical properties of the material.

**Figure 1:**
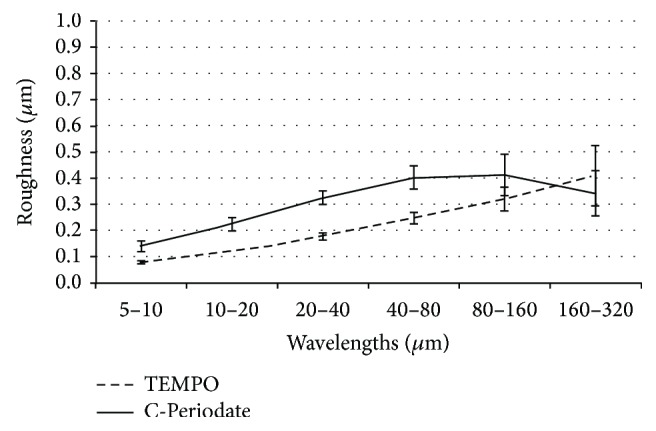
Roughness analysis of the nanocellulose films assessed in this study.

In this study, LP was applied for the surface topography quantification of films made from the TEMPO and C-Periodate nanocelluloses (Figure 1). The surface roughness assessed at various wavelengths yielded an estimation of the fibrillation of the nanocellulose materials. Note that the roughness was relatively low and the assessed samples had levels compared to well-fibrillated and structurally homogeneous nanocelluloses [13, 24]. However, the increase in roughness at wavelengths larger than 40 *μ*m indicated that the nanocelluloses also had a minor fraction of residual fibers. The global roughness of the samples was 0.64 (±0.06) and 1.03 (±0.1) *μ*m for the TEMPO and C-Periodate nanocelluloses, respectively. Further analysis based on a relationship between the roughness of films and the amount of residual fibers [24] indicated that the TEMPO and C-Periodate samples had a fraction of approximately 0.79% and 2.64% residual fibers, respectively.

**Figure 2:**
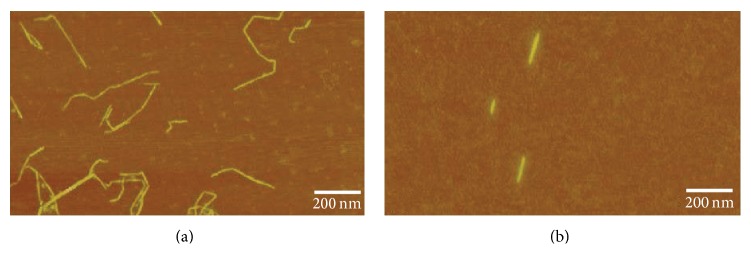
AFM images of cellulose nanofibrils. (a) TEMPO nanofibrils utilized for making the film substrate. (b) C-Periodate nanofibrils for application as a bioink.

Figure 2 shows AFM images which reveal the morphology of the nanofibrils applied in the substrate films (TEMPO) and in bioinks (C-Periodate). Note the major difference with respect to the length of the individual nanofibrils. Carboxymethylation and periodate oxidation yielded short nanofibrils, compared to the TEMPO quality. The lengths of the C-Periodate nanofibrils were less than 200 nm. Also it is important to mention that the estimation of a low fraction of residual fibers confirmed the high structural homogeneity of the nanomaterials.

**Figure 3:**
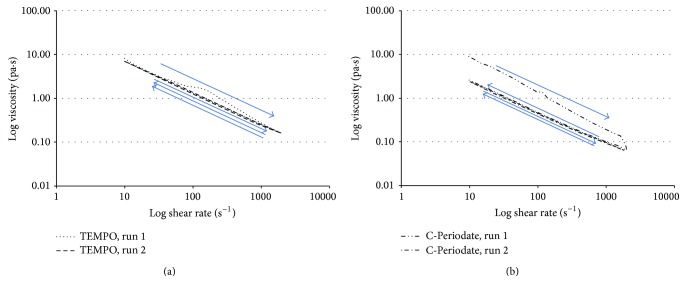
Rheological properties of the TEMPO (a) and C-Periodate (b) nanocelluloses. The increasing and decreasing shear are exemplified with blue arrows.

Additionally, the rheology of the different nanocellulose gels used to make the substrates and deposit 3D constructs was assessed using a simple shear experiment (Figure 3). Simply, the strain rate applied to the material was varied, and the corresponding viscosity was measured. The tests were conducted up to a strain rate of 1000/s. This is similar in magnitude to the strain rate that may be encountered in the extrusion needle where, typically, values up to 2000/s were used to form some samples. Further to minimize the occurrence of slip, the gap between the cone and plate was set to be zero; that is, the apex of the cone was in contact with the plate, and the chosen cone had a diameter of 40 mm and a film angle of 4 degrees. More in-depth rheological studies based on oscillatory methods to explore elastic and viscous components as well as gel point sensitivity will be carried out in future work.

In this study, the nanofibril morphology varied greatly between the two suspensions. However, it can be seen that both suspensions followed the fluid power law with hysteresis between shear rate sweeps. However, upon investigation of a second run, which immediately followed the first, we can see that the viscosity profile is stable after the initial sweep from 1 to 1000 s^−1^. Considering the TEMPO nanocellulose, a similar behavior has been previously reported for fibrillated materials produced without chemical pretreatment [26]. However, the gradient of the C-Periodate is higher than that of the TEMPO quality, suggesting a more pronounced shear thinning, which could be useful for coating and printing applications. It seems that the C-Periodate, which has shorter nanofibrils, is more prone to being broken down under the high strain rates and that is probably the cause of the major hysteresis that is detected. A similar thixotropic behaviour has been reported for cellulose nanocrystals [27], which have a morphology similar to the C-Periodate sample.

**Figure 4:**
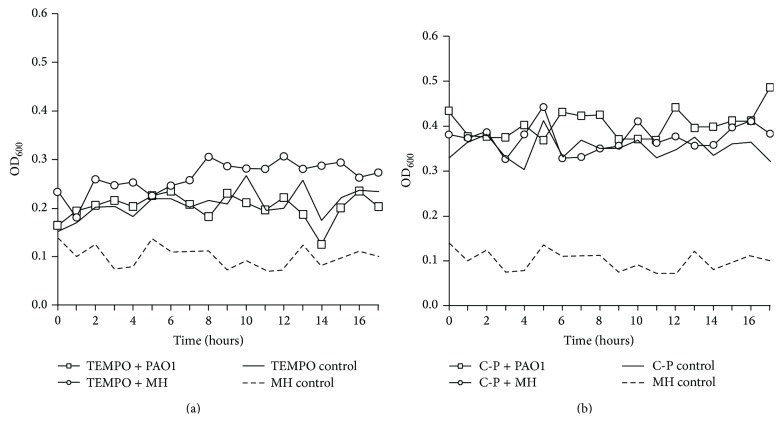
Optical density measurements for the nanocellulose suspensions with/without* P. aeruginosa* PAO1 to determine whether they support or inhibit bacterial growth. (a) TEMPO nanocellulose. (b) C-Periodate nanocellulose.

In these experiments, we also studied the ability of these prototypical nanocellulose materials to support or inhibit the bacterial growth of the common wound pathogen* P. aeruginosa.* The nanocellulose materials with/without MH broth did not show any significant increases in OD_600_ which indicated that the suspensions were free from bacterial contamination (Figure 4). This study also showed that the OD_600_ measurement of the two nanocelluloses when inoculated with* P. aeruginosa* PAO1 did not significantly increase, which indicated that the materials inhibited (did not support) bacterial growth (Figure 4). This inhibition of bacterial growth reveals an interesting property of these novel materials.

**Figure 5:**
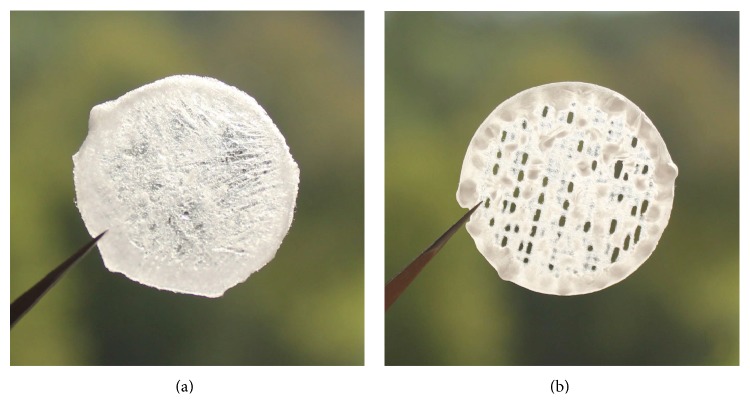
Grid constructs printed with a Bioplotter unit. (a) TEMPO nanocellulose. (b) C-Periodate nanocellulose.

Having clarified the surface chemistry, morphology, rheology, and the ability of the nanocellulose materials to inhibit PAO1 growth, we assessed the suitability of the material to be used as a bioink in 3D printing.  Figure 5 demonstrates two grid constructs using the TEMPO and C-Periodate nanocellulose as bioinks. Contrary to the TEMPO nanocellulose, the C-Periodate nanocellulose formed a more solid structure with defined tracks, whilst the TEMPO nanocellulose tended to collapse probably due to the low consistency of the material (0.95%). The C-Periodate nanocellulose had shorter fibrils, higher consistency (3.9%), and an appropriate rheology which was suitable for being used as a bioink (Figure 3).

**Figure 6:**
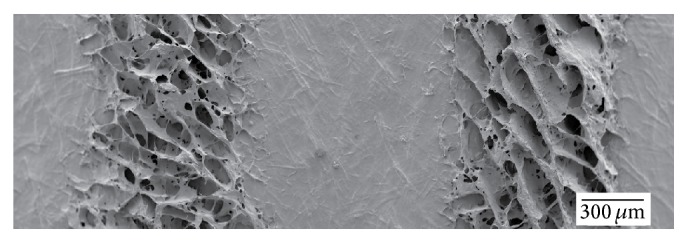
SEM image of porous 3D tracks printed on the surface of a smooth nanocellulose film. The porous tracks can potentially be applied as bioresponsive structures for the controlled release of integrated components.

Figure 6 shows an SEM image of a simple grid construct where the C-Periodate nanocellulose was deposited on a TEMPO nanocellulose film. The deposited material gave a highly porous structure. Such structures have been reported to be highly absorbent and pH-responsive [13]. Hence, similar characteristics can be applied in tailor-made wound dressings with the potential to carry and release antimicrobial components.

**Figure 7:**
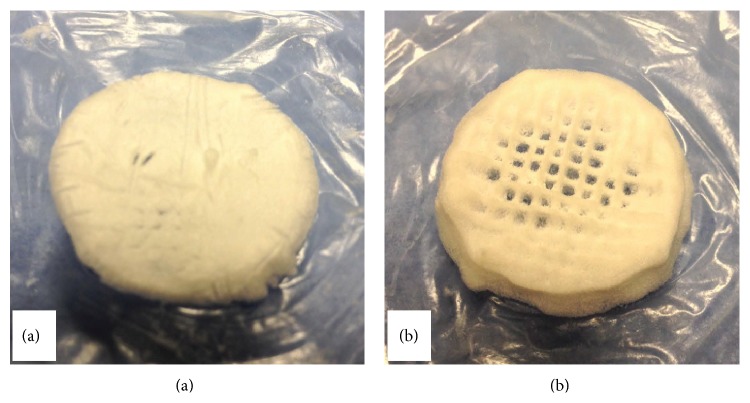
3D printed nanocellulose structures. (a) C-Periodate nanocellulose cross-linked with CaCl_2_ printed on a nanocellulose film. (b) C-Periodate nanocellulose without cross-linking, printed on a nanocellulose film. The dimensions of the printed structures were approximately 25 mm in diameter and 6 mm in height. Nine layers were printed, each consisting of 12 × 12 tracks.

Through appropriate choice of extrusion and movement speed, the Bioplotter was also used to explore the possibility to produce a fully 3D construct composed of nine layers based on the C-Periodate nanocellulose, as shown in Figure 7. Subsequent to deposition, freeze-drying produced self-standing porous structures that can also be applied as scaffolds for tissue regeneration. Figure 7 shows the printed nanocellulose scaffolds photographed after freeze-drying. During the printing, some of the scaffolds were cross-linked with CaCl_2_ (Figure 7(a)).  Figure 7(b) shows a scaffold that was not cross-linked with CaCl_2_ during the printing. This demonstrates that bioplotting has the potential to form well-defined tracks in a self-standing 3D structure. It is worth mentioning that attempts to use TEMPO nanocellulose as a bioink resulted in 3D structures that collapsed and no defined tracks could be observed. This was probably due to the low consistency of the TEMPO nanocellulose (0.95%). However, due to the relatively high viscosity of the TEMPO nanocellulose, it was not possible to increase the consistency by centrifugation, as was the case with the C-Periodate sample.

Figure 7 shows that CaCl_2_ had an adverse effect on the appearance of the scaffold, obscuring the “macro” pores made by the 3D plotter. This effect was seen only after freeze-drying the samples. CaCl_2_ stabilized the structures by forming ionic links between the carboxyl groups (COO^−^) and the divalent cations (Ca^2+^) [13]. The CaCl_2_ was applied by simply pipetting the solution into the printed nanocellulose structure. These factors seem to have compacted the structure (Figure 7(a)). However, exploration of this approach demonstrates some promise and further trials are required involving refinement of the bioplotting sequence and a more controlled application of the CaCl_2_ cross-linker.

Although calcium nanocellulose films have not yet been tested in wound management, wound healing has been shown to be a calcium-mediated process, where it is involved in a number of signalling cascades important in healing the wound [28, 29]. Calcium alginate dressings are routinely used clinically and numerous studies have found that these dressings increase the rate of wound healing [29, 30]. Therefore, the inclusion of calcium salts in nanocellulose dressing materials is not contraindicated.

## 4. Conclusions

This study shows how nanocellulose may be formulated to manufacture substrates and gels that may be deposited as a bioink through a printing process. Based on the printing of grid structures, we conclude that the C-Periodate nanocellulose was suitable for being used as bioink. This was most probably due to the higher consistency and appropriate rheology of the C-Periodate nanocellulose achieved in this study. The nanocelluloses form 3D structures where the tracks have an open porosity and the potential to carry and release antimicrobial components. We demonstrated that the nanocelluloses assessed in this study did not support bacterial growth. The properties of these materials will be a distinct advantage for wound dressing applications.

## Figures and Tables

**Table 1:** DP and surface chemistry of the nanocelluloses applied in this study.

Code	DP	Aldehyde content (*μ*mol/g)	Carboxyl content (*μ*mol/g)
TEMPO	709	71	855
C-Periodate^*^	<80	1202	393

^*∗*^Values from [13].

## References

[1] Saito T., Nishiyama Y., Putaux J.-L., Vignon M., Isogai A. (2006). Homogeneous suspensions of individualized microfibrils from TEMPO-catalyzed oxidation of native cellulose. *Biomacromolecules*.

[2] Pääkko M., Ankerfors M., Kosonen H. (2007). Enzymatic hydrolysis combined with mechanical shearing and high-pressure homogenization for nanoscale cellulose fibrils and strong gels. *Biomacromolecules*.

[3] Wågberg L., Decher G., Norgren M., Lindström T., Ankerfors M., Axnäs K. (2008). The build-up of polyelectrolyte multilayers of microfibrillated cellulose and cationic polyelectrolytes. *Langmuir*.

[4] Saito T., Hirota M., Tamura N. (2009). Individualization of nano-sized plant cellulose fibrils by direct surface carboxylation using TEMPO catalyst under neutral conditions. *Biomacromolecules*.

[5] Syverud K., Chinga-Carrasco G., Toledo J., Toledo P. G. (2011). A comparative study of *Eucalyptus* and *Pinus radiata* pulp fibres as raw materials for production of cellulose nanofibrils. *Carbohydrate Polymers*.

[6] Jonoobi M., Mathew A. P., Oksman K. (2012). Producing low-cost cellulose nanofiber from sludge as new source of raw materials. *Industrial Crops and Products*.

[7] Alila S., Besbes I., Vilar M. R., Mutjé P., Boufi S. (2013). Non-woody plants as raw materials for production of microfibrillated cellulose (MFC): a comparative study. *Industrial Crops and Products*.

[8] Henriksson M., Berglund L. A., Isaksson P., Lindström T., Nishino T. (2008). Cellulose nanopaper structures of high toughness. *Biomacromolecules*.

[9] Fukuzumi H., Saito T., Iwata T., Kumamoto Y., Isogai A. (2009). Transparent and high gas barrier films of cellulose nanofibers prepared by TEMPO-mediated oxidation. *Biomacromolecules*.

[10] Chinga-Carrasco G., Tobjörk D., Österbacka R. (2012). Inkjet-printed silver nanoparticles on nano-engineered cellulose films for electrically conducting structures and organic transistors: concept and challenges. *Journal of Nanoparticle Research*.

[11] Orelma H., Filpponen I., Johansson L.-S., Österberg M., Rojas O. J., Laine J. (2012). Surface functionalized nanofibrillar cellulose (NFC) film as a platform for immunoassays and diagnostics. *Biointerphases*.

[12] Way A. E., Hsu L., Shanmuganathan K., Weder C., Rowan S. J. (2012). pH-responsive cellulose nanocrystal gels and nanocomposites. *ACS Macro Letters*.

[13] Chinga-Carrasco G., Syverud K. (2014). Pretreatment-dependent surface chemistry of wood nanocellulose for pH-sensitive hydrogels. *Journal of Biomaterials Applications*.

[14] Vartiainen J., Pöhler T., Sirola K. (2011). Health and environmental safety aspects of friction grinding and spray drying of microfibrillated cellulose. *Cellulose*.

[15] Alexandrescu L., Syverud K., Gatti A., Chinga-Carrasco G. (2013). Cytotoxicity tests of cellulose nanofibril-based structures. *Cellulose*.

[16] Dong S., Hirani A. A., Colacino K. R., Lee Y. W., Roman M. (2012). Cytotoxicity and cellular uptake of cellulose nanocrystals. *Nano LIFE*.

[17] Ang T. H., Sultana F. S. A., Hutmacher D. W. (2002). Fabrication of 3D chitosan-hydroxyapatite scaffolds using a robotic dispensing system. *Materials Science and Engineering C*.

[18] Landers R., Hübner U., Schmelzeisen R., Mülhaupt R. (2002). Rapid prototyping of scaffolds derived from thermoreversible hydrogels and tailored for applications in tissue engineering. *Biomaterials*.

[19] Wang M.-D., Zhai P., Schreyer D. J. (2013). Novel crosslinked alginate/hyaluronic acid hydrogels for nerve tissue engineering. *Frontiers of Materials Science*.

[20] Billiet T., Gevaert E., de Schryver T., Cornelissen M., Dubruel P. (2014). The 3D printing of gelatin methacrylamide cell-laden tissue-engineered constructs with high cell viability. *Biomaterials*.

[21] Inzana J. A., Olvera D., Fuller S. M. (2014). 3D printing of composite calcium phosphate and collagen scaffolds for bone regeneration. *Biomaterials*.

[22] Saito T., Isogai A. (2004). TEMPO-mediated oxidation of native cellulose. The effect of oxidation conditions on chemical and crystal structures of the water-insoluble fractions. *Biomacromolecules*.

[23] ISO (2010). Pulps—determination of limiting viscosity number in cupri-ethylenediamine (CED) solution. *ISO*.

[24] Chinga-Carrasco G., Averianova N., Kondalenko O. (2014). The effect of residual fibres on the micro-topography of cellulose nanopaper. *Micron*.

[25] Fukuzumi H., Saito T., Isogai A. (2013). Influence of TEMPO-oxidized cellulose nanofibril length on film properties. *Carbohydrate Polymers*.

[26] Iotti M., Gregersen Ø. W., Moe S., Lenes M. (2011). Rheological studies of microfibrillar cellulose water dispersions. *Journal of Polymers and the Environment*.

[27] Hasani M., Cranston E. D., Westman G., Gray D. G. (2008). Cationic surface functionalization of cellulose nanocrystals. *Soft Matter*.

[28] Boateng J. S., Matthews K. H., Stevens H. N. E., Eccleston G. M. (2008). Wound healing dressings and drug delivery systems: a review. *Journal of Pharmaceutical Sciences*.

[29] Kawai K., Larson B. J., Ishise H. (2011). Calcium-based nanoparticles accelerate skin wound healing. *PLoS ONE*.

[30] Doyle J. W., Roth T. P., Smith R. M., Li Y.-Q., Dunn R. M. (1996). Effect of calcium alginate on cellular wound healing processes modeled in vitro. *Journal of Biomedical Materials Research*.

